# Proteomics early after heart transplantation and relation to coronary intimal changes and prognosis

**DOI:** 10.1016/j.jhlto.2024.100110

**Published:** 2024-05-13

**Authors:** Rasmus Gebauer Dalsgaard, Tor Skibsted Clemmensen, Hans Eiskjær, Steen Hvitfeldt Poulsen, Kamilla Pernille Bjerre

**Affiliations:** a Department of Cardiology, Aarhus University Hospital, Aarhus, Denmark; b Department of Clinical Medicine, Faculty of Health, Aarhus University, Aarhus, Denmark; c Department of Internal Medicine, Regional Hospital Horsens, Horsens, Denmark

**Keywords:** heart transplantation, cardiac allograft vasculopathy, optical coherence tomography, proteomics, intravascular imaging

## Abstract

**Background:**

Long-term survival after heart transplantation (HTx) is limited by cardiac allograft vasculopathy (CAV). The pathogenesis of CAV is poorly understood, and treatment has not yet been well-established. In this exploratory study, we aimed to identify novel immune and nonimmune biomarkers correlated to CAV development, and biomarkers predicting CAV-related events.

**Methods:**

Using a proteomic panel, 92 cardiovascular disease-related proteins were evaluated in 26 de novo HTx patients at 3 and 12 months post-transplantation. Intima-area changes were assessed using optical coherence tomography. Major adverse cardiac events (MACE) included significant CAV progression, heart failure, and cardiovascular death.

**Results:**

The median follow-up was 6.8 years. We found changes in 4 inflammatory biomarkers: matrix metalloproteinase 3 (MMP-3) and matrix metalloproteinase 2 (MMP-2) were significantly increased after 3 months in the group of patients with the highest intima proliferation, and after 12 months in patients experiencing MACE, respectively. Monocyte chemotactic protein 1 (MCP-1) was increased after 12 months in patients experiencing MACE. Platelet-derived growth factor subunit A (PDGF-A) was significantly increased after 3 months in the group of patients with highest intima proliferation.

**Conclusions:**

We identified 4 biomarkers that may be associated with CAV development which differed significantly between groups of intima proliferation and MACE; MMP-2, MMP-3, MCP-1, and PDGF-A. Further studies are needed to examine whether our findings offer the basis to seek new markers of graft vasculopathy or treatment options.

## Background

Heart transplantation (HTx) remains the best treatment for selected patients with end-stage heart failure. However, long-term survival after HTx is limited mainly by cardiac allograft vasculopathy (CAV), which affects approximately 50% of HTx patients 10 years after transplantation.^1^

CAV is a generalized form of cardiac vascular disease affecting epicardial coronary arteries, microcirculation, and coronary veins. It is characterized by diffuse, concentric lesions with intimal proliferation due to the accumulation of smooth muscle cells (SMC) and the fibrous matrix they produce.^1^, ^2^ CAV is often clinically silent due to the denervation of the transplanted hearts but can affect the graft function and lead to heart failure-related symptoms. The pathogenesis of CAV is poorly understood, and the development is caused by both immunological and nonimmunological factors. Both cellular and antibody-mediated rejections increase the risk of developing CAV.^2^, ^3^ The presence of donor-specific antibodies has been shown to be associated with significantly increased CAV burden, microvascular dysfunction, and restrictive physiology.^3^

Treatment of CAV has not yet been well-established. In some cases, percutaneous coronary intervention may serve as treatment, and selected patients may benefit from retransplantation. While mammalian target of rapamycin (mTOR) inhibitors have demonstrated a preventive effect if initiated early,^4^ it remains uncertain, which patients will derive benefit from this treatment. The scarce treatment options for CAV may be related to the incompletely understood pathogenesis. Therefore, knowledge about immune and nonimmune pathways associated with CAV may pave the way for future treatment.

In this exploratory study, we aim to identify novel immune and nonimmune biomarkers correlated to CAV development, and to identify biomarkers that provide prognostic information in relation to CAV-related events.

## Methods

### Study population

The study population comprised 29 patients who underwent HTx at Aarhus University Hospital from July 2013 through January 2016. Three patients died during follow-up. Thus, blood samples and optical coherence tomography (OCT) scans of coronary arteries were available in 26 patients at 3 and 12 months after HTx. All patients were ≥18 years of age, had a creatinine level <200 µmol/liter, and provided informed written consent in accordance with the Helsinki Declaration principles. The study was approved by the Central Denmark Region Committees on Biomedical Research Ethics and was registered with ClinicalTrials.gov (NCT02077764). Data from OCT scans have been presented in an earlier study.^5^

### Post-transplant care and follow-up

All patients received antithymocyte globulin as induction therapy, and the initial immunosuppressive regimen included prednisolone, tacrolimus, and mycophenolate mofetil. Pravastatin was routinely started in all patients. Patients did not routinely commence aspirin therapy as primary prophylaxis; it was initiated only in the presence of CAV or on noncardiac indication.

Routine CAV surveillance comprised coronary angiography (CAG) at 3 and 12 months after HTx followed by CAG either annually or every second year.

The patients underwent routine endomyocardial biopsies for rejection surveillance during the first 2 postoperative years. Biopsies were planned weekly for the first 6 weeks, then at 2-week intervals until the third month, followed by monthly intervals until the sixth month. Subsequently, biopsies were scheduled every 2 months for the remainder of the first year and afterward every 3 months until the end of the second year. Afterward, biopsies were only performed in cases with clinically suspected rejection. Graft function was assessed by echocardiography on the same day of biopsies and/or CAG.

We retrospectively assessed major adverse cardiac events (MACE) using electronic patient records from OCT at 12 months until censoring on September 26, 2023. MACE included (1) hospitalization due to heart failure defined as the need for intravascular diuretics or inotropes, (2) significant CAV progression defined as an increment in International Society of Heart and Lung Transplantation CAV grading, percutaneous coronary intervention, or occluded vessel without intervention, or (3) cardiovascular death.

### Optical coherence tomography

OCT recordings were acquired 3 and 12 months after HTx using Lunawave OCT (Terumo, Japan) aiming for the longest possible pullbacks up to 150 mm of each major branch including left main coronary artery. Pullback speed was adjusted to obtain a scan time of 3 to 4 seconds while flushing with 15 to 20 ml of contrast.

A quantitative cross-sectional matched analysis with a longitudinal sampling frequency of approximately 1 mm was conducted by a single investigator (T.S.C.) in a previous study^5^ using a customized version of the QCU-CMS analysis software (Leiden University Medical Centre, Leiden, The Netherlands), which has been previously validated. Assessment of vessel layers involved lumen, intima, and media area derived from 3 distinct vessel contours: lumen-intima, intima-media, and media-adventitia interface.

In this study, only left anterior descending artery (LAD) pullbacks were used, as we have previously shown that LAD is more affected by CAV pathophysiology than the left circumflex artery (LCX) and the right coronary artery, and that OCT markers of CAV from LAD were superior to LCX and right coronary artery in predicting disease progression.^6^ In 1 patient, recording of LAD was not possible, and therefore, LCX was used.

### Blood sampling and proteomics

Blood samples were drawn from the cubital vein in relation to OCT recordings and were anticoagulated with sodium citrate. Before proximity extension assay analysis, blood samples were obtained in 1.5 ml tubes, centrifugated at 3,000*g* for 25 minutes at 20°C and stored at −80°C.

Plasma sample analyses were performed by BioXpedia A/S, Aarhus, Denmark, using 1 µl of citrate plasma for the commercial biomarker panel CARDIOVASCULAR III (Olink Bioscience). The panel is based on a proximity extension assay technique providing 92 cardiovascular disease-related proteins. It contains both known nonimmune cardiovascular markers, exploratory human proteins with the potential of being new cardiovascular disease markers as well as inflammatory and immune markers.

Ninety-two oligonucleotide-labeled antibody-probe pairs (proximity extension assay probes) were incubated with the plasma samples, and these bind to their target proteins when reaching proximity so hybridization occurs. To quantify the biomarkers, a microfluid real-time quantitative polymerase chain reaction (96.96 Dynamic Array Integrated Fluidic Circuit Fluidigm BioMark) was used after adding DNA polymerase to generate templates for DNA amplification.

All biomarker values are expressed in an arbitrary measuring unit, Normalized Protein eXpression on a log2 scale. High Normalized Protein eXpression values correspond to high protein concentrations.

### Statistical analyses

This is an exploratory study. To minimize multiple significance tests, we predefined and limited our outcome measures to MACE and intima area at 3 and 12 months after transplantation, considering the nature of CAV as an intimal proliferative disease. We calculated delta values in intima area from 3 to 12 months and divided the patients into 2 groups; above and below the median. Furthermore, we divided the patients into 2 groups according to MACE in the period between 12 months post-transplant until end of follow-up. Patients were censored at first MACE.

Statistical analyses were performed using GraphPad Prism 8.4.2 (GraphPad Software Inc., San Diego, CA). Normal distribution of continuous data was checked visually using quantile-quantile-plots in STATA 18.0 (StataCorp, College Station, TX). When assessing between-group differences, unpaired *t*-test was used for normally distributed data presented as mean ± standard deviation. Nonnormally distributed data were assessed using Wilcoxon rank sum test and are presented as median and interquartile range (IQR).

All tests were 2-sided, and *p*-values <0.05 were considered statistically significant. We used a multivariable regression model to assess the independent significance of biomarkers with *p*-values <0.05 in the univariate analysis.

Only biomarkers with *p*-values <0.01 in the univariate analysis and statistically significant biomarkers in more than 1 group considered relevant for the CAV pathogenesis will be reviewed in the Discussion.

## Results

### Patient demographics

Patient characteristics as well as routine biochemistry at baseline are shown in Table 1.

**Table 1:** Baseline Patient Characteristics of 26 Heart Transplant Recipients

Characteristics	Baseline characteristics (*N* = 26 patients)
Men, *n* (%)	21 (81)
Age at HTx (years)	55 [40-63]
Reason for HTx	
Cardiomyopathy [*n* (%)]	15 (58)
Ischemic heart disease [*n* (%)]	6 (23)
Other [*n* (%)]	5 (19)
Cold ischemic time (minutes)	182 ± 48
Donor age (years)	48 [37-53]
Diabetes (%)	4 (15)
Hypertension (%)	18 (69)
Medication	
Prednisolone [*n* (%)]	26 (100)
Tacrolimus [*n* (%)]	26 (100)
Everolimus [*n* (%)]	3 (12)
Mycophenolate mofetil [*n* (%)]	26 (100)
Statins [*n* (%)]	23 (88)
ACE/AT-II inhibitor [*n* (%)]	11 (42)
Loop diuretics [*n* (%)]	5 (19)
Calcium channel blocker [*n* (%)]	5 (19)
Aspirin [*n* (%)]	3 (12)
Biochemistry	
Creatinine (µmol/liter)	101 [82-117]
Hemoglobin (mmol/liter)	7.8 [7.1-8.4]
Total cholesterol (mmol/liter)	4.9 ± 1.2
Troponin T (ng/liter)	24 [17-35]
NT-proBNP (ng/liter)	674 [463-1031]

Abbreviations: ACE, angiotensin-converting enzyme; AT-II, angiotensin II; HTx, heart transplantation; NT-proBNP, N-terminal probrain natriuretic peptide.

Data presented as number (%), median [interquartile range] or mean ± standard deviation.

The 26 de novo HTx patients had a median age of 55 (IQR 40-63) years. The majority of the patients were males who underwent transplantation due to cardiomyopathy.

Median follow-up was 6.8 (IQR 3.8-8.5) years. A total of 15 patients (58%) reached a MACE end-point with a median time to MACE at 4.4 (IQR 1.0-6.5) years. Reasons for censoring were CAV progression (*n* = 9), heart failure admission (*n* = 4), and cardiovascular death (*n* = 2). Of these patients, 6 experienced acute cellular rejection episodes ≥2R during follow-up.

### Optical coherence tomography

**Figure 1:**
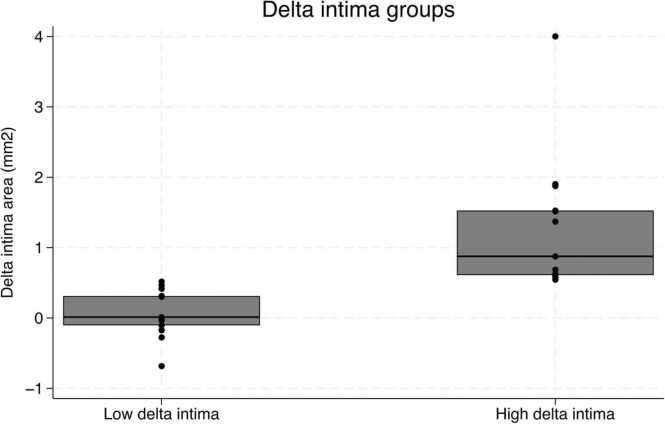
The patients were divided into 2 groups based on intima proliferation, categorized as low vs high delta intima, respectively.

Twenty-six vessels were available for OCT analysis at 3 and 12 months, respectively. Median intima area increased 0.53 [IQR 0.01-0.88] mm^2^. This value served as a cut-off point for dividing patients into 2 groups based on intima proliferation (high and low delta intima). These 2 groups are depicted in Figure 1.

### Biomarkers

All 92 biomarkers were analyzed successfully. A total of 23 biomarkers differed significantly between intima area groups assessed by OCT (Table 2) and between MACE groups (Table 3), respectively. Spreadsheets of the raw data can be found in the Supplementary.

**Table 2:** Statistically Significant Biomarkers Assessed by Unpaired *t*-Tests After 3 and 12 Months, Respectively, in Patients Dichotomized From the Change in Intima Area From 3 to 12 Months According to OCT Scans

Biomarkers	Low delta intima (*n* = 13)	High delta intima (*n* = 13)	*p*-value	Adjusted *p*-value
3 Months biomarker analyses				
Low-density lipoprotein receptor	3.36 (±0.37)	4.02 (±0.65)	0.004	0.57
Integrin beta-2	3.96 (±0.32)	4.32 (±0.44)	0.023	0.33
Epithelial cell adhesion molecule	4.27 (±0.66)	5.00 (±0.82)	0.019	0.94
Matrix metalloproteinase 3	8.37 (±0.84)	9.10 (±0.63)	0.020	0.62
Platelet-derived growth factor subunit A	4.51 (±1.08)	5.48 (±0.46)	0.006	0.06
Myeloblastin	5.37 (±0.62)	4.78 (±0.65)	0.027	0.09
12 Months biomarker analyses				
Protein delta homolog 1	5.34 (±0.55)	5.96 (±0.61)	0.012	0.10
Tumor necrosis factor receptor superfamily member 6	5.53 (±0.25)	5.75 (±0.27)	0.047	0.20
Trem-like transcript 2 protein	5.15 (±0.29)	4.79 (±0.35)	0.008	0.38
Myeloperoxidase	3.65 (±0.37)	3.26 (±0.35)	0.011	0.78
Myeloblastin	4.63 (±0.65)	4.06 (±0.54)	0.023	0.74
Matrix extracellular phosphoglycoprotein	5.74 (±0.46)	5.29 (±0.46)	0.020	0.02

Abbreviation: OCT, optical coherence tomography.

Data are presented as mean ± standard deviation.

We employed a multivariable regression model to evaluate the independent significance of biomarkers that exhibited *p-values <0.05 in the univariate analysis.*

**Table 3:** Statistically Significant Biomarkers Assessed by Unpaired *t*-Tests After 3 and 12 Months, Respectively, in Patients Dichotomized According to MACE End-Points

Biomarkers	No MACE (*n* = 11)	MACE (*n* = 15)	*p*-value	Adjusted *p*-value
3 Months biomarker analyses				
Trem-like transcript 2 protein	5.15 (±0.32)	4.81 (±0.45)	0.047	-
12 Months biomarker analyses				
P-selectin	9.62 (±0.62)	10.1 (±0.49)	0.047	0.65
Monocyte chemotactic protein 1	4.51 (±0.50)	5.21 (±0.34)	<0.001	0.28
Galectin-3	2.82 (±0.26)	3.08 (±0.32)	0.044	0.87
Bleomycin hydrolase	1.95 (±0.36)	2.41 (±0.43)	0.008	0.56
Fatty acid-binding protein—adipocyte	5.25 (±0.73)	5.92 (±0.82)	0.040	0.39
Matrix metalloproteinase 2	3.05 (±0.22)	3.31 (±0.34)	0.036	0.02
Cathepsin D	2.65 (±0.25)	3.00 (±0.44)	0.027	0.50
Carboxypeptidase A1	5.43 (±0.68)	6.10 (±0.75)	0.030	0.75
Carboxypeptidase B	5.05 (±0.48)	5.68 (±0.74)	0.021	0.77
Tissue-type plasminogen activator	6.57 (±0.48)	7.24 (±0.57)	0.004	0.71
Cathepsin Z	4.93 (±0.17)	5.21 (±0.33)	0.017	0.56
Retinoic acid receptor responder protein 2	11.3 (±0.27)	11.5 (±0.25)	0.042	0.21

Abbreviations: MACE, major adverse cardiac events.

Data are presented as mean ± standard deviation.

We employed a multivariable regression model to evaluate the independent significance of biomarkers that exhibited *p-values <0.05 in the univariate analysis.*

### Biomarkers related to intima area increase

Three months after HTx, patients with high delta intima had significantly higher values of 5 biomarkers compared with patients with low delta intima; low-density lipoprotein receptor, integrin beta-2, epithelial cell adhesion molecule, matrix metalloproteinase 3 (MMP-3), and platelet-derived growth factor subunit A (PDGF-A) (all, *p* < 0.05). Myeloblastin (PRTN3) was significantly lower in patients with high delta intima compared with patients with low delta intima (*p* < 0.05).

Twelve months after HTx, 2 biomarkers were significantly increased in patients with high delta intima compared with patients with low delta intima; protein delta homolog 1 and tumor necrosis factor receptor superfamily member 6 (both, *p* < 0.05), whereas 4 biomarkers were significantly lower; trem-like transcript 2 protein (TLT-2), myeloperoxidase, myeloblastin (PRTN3), and matrix extracellular phosphoglycoprotein (all, *p* < 0.05).

**Figure 2:**
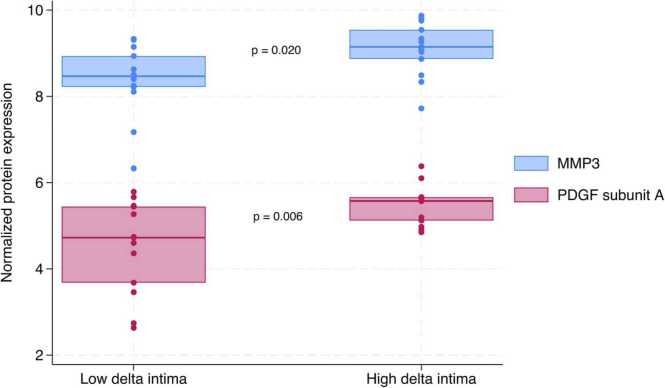
Boxplots depicting MMP-3 (*p* = 0.020) and PDGF-A (*p* = 0.006) levels after 3 months in patients with low vs high delta intima. MMP-3, matrix metalloproteinase 3; PDGF-A, platelet-derived growth factor subunit A.

Biomarkers reviewed in the discussion are illustrated in Figure 2, while the remaining biomarkers are shown in the Supplementary.

### Biomarkers related to MACE

In patients experiencing MACE, TLT-2 was significantly decreased 3 months after HTx compared with patients who did not experience MACE (*p* < 0.05).

A total of 12 biomarkers were significantly increased 12 months after HTx in patients experiencing MACE compared with patients who did not experience MACE; P-selectin, monocyte chemotactic protein 1 (MCP-1), galectin-3, bleomycin hydrolase, fatty acid-binding protein—adipocyte, matrix metalloproteinase 2 (MMP-2), cathepsin D, carboxypeptidase A1, carboxypeptidase B, tissue-type plasminogen activator, cathepsin Z, and retinoic acid receptor responder protein 2 (all, *p* < 0.05).

**Figure 3:**
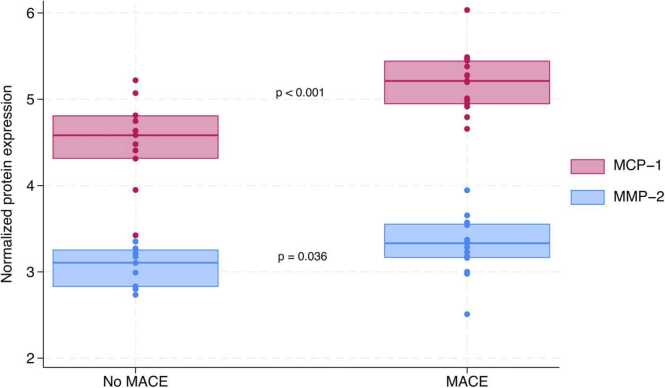
Boxplots depicting MCP-1 (*p* < 0.001) and MMP-2 (*p* = 0.036) levels after 12 months in patients experiencing MACE vs no MACE. MACE, major adverse cardiac events; MCP-1, monocyte chemotactic protein 1; MMP-2, matrix metalloproteinase 2.

Biomarkers reviewed in the discussion are illustrated in Figure 3, while the remaining biomarkers are shown in the Supplementary.

## Discussion

In this explorative study, we used a novel proximity extension assay technique to evaluate 92 cardiovascular biomarkers in HTx patients related to intima proliferation and MACE. Our main findings were (1) 12 biomarkers showed statistically significant differences between low and high delta intima groups and (2) 13 biomarkers showed statistically significant differences between MACE groups.

The metalloproteinases MMP-3 and MMP-2 were significantly increased after 3 months in patients with high delta intima and after 12 months in patients experiencing MACE, respectively. TLT-2 was significantly decreased after 12 months in patients with high delta intima and after 3 months in patients experiencing MACE, respectively. PRTN3 was significantly increased after 3 and 12 months in patients with low delta intima.

Of the biomarkers that differed significantly between groups, low-density lipoprotein receptor, PDGF-A, MCP-1, bleomycin hydrolase, and tissue-type plasminogen activator had *p*-values <0.01. We focused on the biomarkers with biological roles in line with the proposed CAV pathogenesis as described in the literature; MMP-2 and MMP-3, MCP-1 and PDGF-A.

### Inflammatory biomarkers

The metalloproteinases are well-described as being involved in the development of atherosclerosis, and they have been described in relation to CAV, as well. During SMC proliferation, degradation and/or remodeling of the extracellular matrix (ECM) is required. Metalloproteinases are responsible for ECM degradation, each having a specific function.^7^ Studies have shown a relation between MMP-2 and ECM turnover and subsequent CAV development. Furthermore, MMP-2 suppression has been shown to attenuate CAV development in a murine model.^7^ Our study showed elevated levels of MMP-2 after 12 months in patients experiencing MACE. Regarding MMP-3, studies have found a direct association between MMP-3 and coronary artery disease, subsequent complications, and clinical outcomes.^8^ Accordingly, our study showed elevated levels after 3 months in patients with high delta intima, suggesting that there might be an association with CAV development.

MCP-1 is a chemokine implicated in endothelial inflammatory processes.^9^ It is secreted by the vascular endothelium and is responsible for the attraction of leukocytes to the site of injury as well as the proliferation and migration of SMCs to the subendothelial space.^10^ Furthermore, MCP-1 has been studied in relation to intima proliferation in CAV. An association between MCP-1-expression, macrophage recruitment, and intimal thickening in a murine model has been established.^11^ In HTx patients with CAV, circulating plasma levels of MCP-1 have also been shown to be increased compared with HTx patients without CAV.^12^ In this study, MCP-1 was increased 12 months after transplantation in patients experiencing MACE. However, we did not find any significant differences between delta intima area groups either 3 or 12 months after transplantation.

A relation between PDGF-A and CAV has been described in several studies. Increased expression has been observed in the pathologic proinflammatory cardiac allograft, as its presence has been related to CAV development^13^ as well as conventional atherosclerosis.^14^ The role of PDGF-A in the development of CAV may be related to the regulation of pathological SMC proliferation and arteriosclerosis in the cardiac allografts,^15^ as the differentiation of vascular progenitor cells into endothelial cells and SMC is regulated by PDGF.^14^ In vitro experiments on human endothelial cells have demonstrated this relation in cardiac allografts, as PDGF-A is expressed in human cardiac allografts but not in normal human hearts. In line, our study found that PDGF-A was significantly increased after 3 months in patients with high delta intima. However, it was not increased after 12 months. This might suggest that PDGF-A is an early initiator of CAV development replaced by other mechanisms in later CAV progression. PDGF-A may be considered as a future therapeutic strategy, and it has already been shown that inhibition of both PDGF and vascular endothelial growth factor is effective in targeting inflammation and pathologic vascular remodeling.^16^

### Clinical implications

The CAV pathophysiology is complex and consists of both immunological and nonimmunological factors. Biomarkers for risk stratifying HTx patients regarding CAV development are desirable but lacking. Furthermore, biochemical pathways in CAV development are not well-described. In this study, we identified various inflammatory biomarkers possibly related to CAV development and CAV-related MACE. These inflammatory pathways may present new therapeutic targets, hopefully paving the way for more personalized treatment. Previous studies have reported promising results regarding anti-inflammatory therapy and a decrease in cardiovascular events among patients with conventional ischemic heart disease.^17^, ^18^ A similar effect could potentially exist in HTx patients with regard to CAV development, and with this study, we hope to pave the way for further research.

### Strengths and limitations

In this exploratory hypothesis-generating biomarker study in de novo HTx patients, we used a novel proteomic panel to identify biomarkers that might be implicated in CAV pathogenesis. To reveal early signs of CAV, state-of-the-art imaging by OCT has been performed, making it possible to detect even small changes in the coronary vessel layers. All patients were followed at our department for approximately 7 years after transplantation. All data were carefully recorded, and no patients were lost to follow-up. Nevertheless, the study also has several limitations that must be taken into consideration. The study is a single-center study with a small study population. The small population challenges the statistics because of the large number of biomarkers employed in the analyses. Multiple testing corrections were performed, and as expected considering the sample size and number of tests, none of the biomarkers reached significance levels, making careful interpretation of the results crucial. Therefore, there is a risk that the findings are the result of type 1 and 2 errors. In accordance with these limitations, we have carefully chosen to use the data as an exploratory study.

## Conclusions

We found significant changes in the inflammatory biomarkers MCP-1, MMP-2, MMP-3, and PDGF-A in patients with high intima proliferation values and patients experiencing MACE. Further studies are needed to examine whether our findings offer the basis to seek new markers of graft vasculopathy or treatment options.

## CRediT authorship contribution statement

**Rasmus Gebauer Dalsgaard:** Writing - original draft, Formal analysis. **Tor Skibsted Clemmensen:** Investigation, Formal analysis, Writing - review and editing. **Hans Eiskjær:** Writing - review andediting. **Steen Hvitfeldt Poulsen:** Writing - review and editing. **Kamilla Pernille Bjerre:** Conceptualization, Funding acquisition, Formal analysis, Writing - review and editing.

## Disclosure statement

The authors declare the following financial interests/personal relationships which may be considered as potential competing interests: Kamilla Pernille Bjerre reports financial support was provided by Aase and Ejnar Danielsens Foundation. The other authors declare that they have no known competing financial interests or personal relationships that could have appeared to influence the work reported in this paper.

We greatly thank all the invasive cardiologists who performed the OCT scans.

The project was funded by the Aase and Ejnar Danielsens Foundation.
