# Supplementary material for: Proteomics early after heart transplantation and relation to coronary intimal changes and prognosis
Source: JHLT Open. 2024 May 13;5:100110. doi: 10.1016/j.jhlto.2024.100110 (PMC11935488; doi:10.1016/j.jhlto.2024.100110)
Supplement: Supplementary file 5 — Supplementary material [file mmc5.docx]

# Appendix B

Illustrations of biomarkers not reviewed in the discussion.

## Figure B1


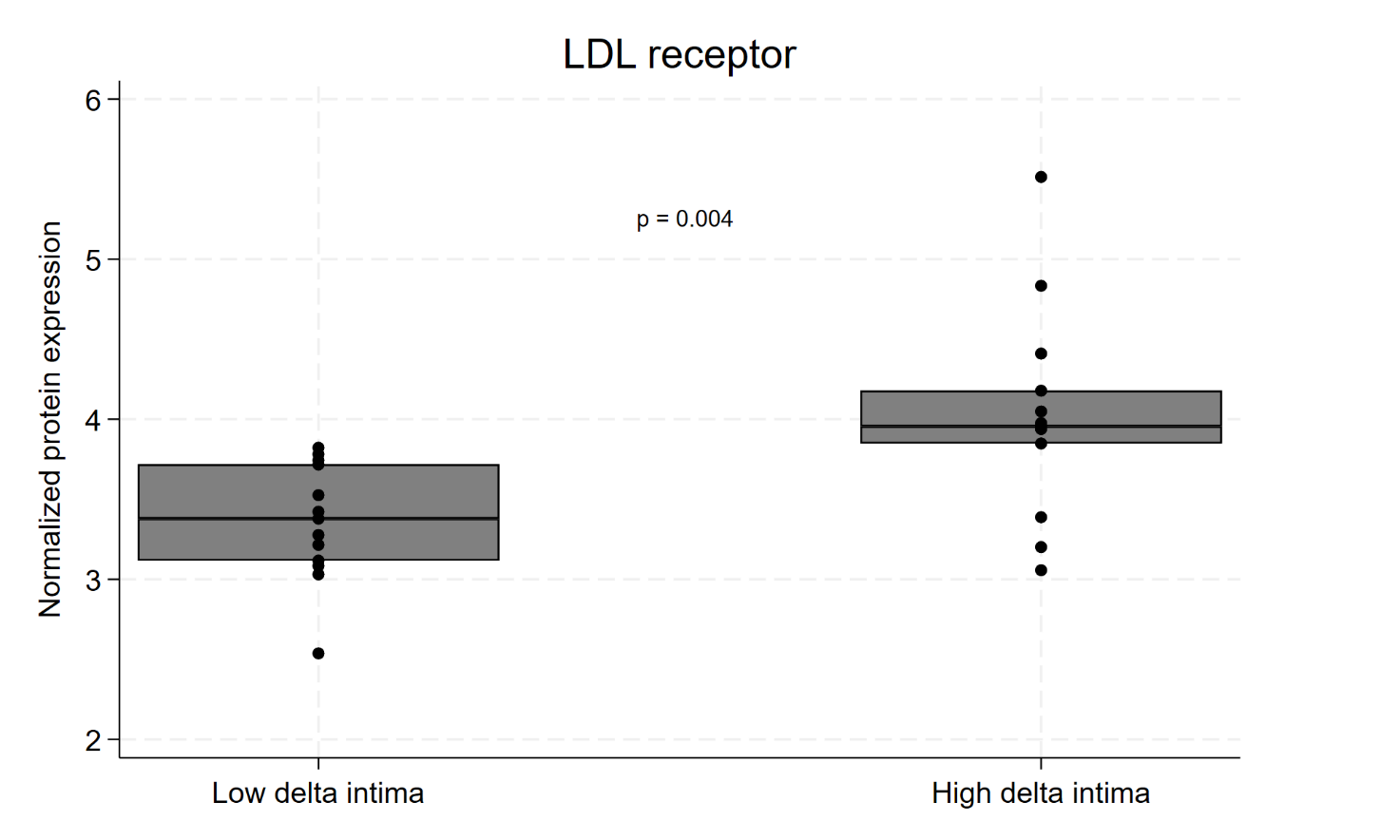


*Figure B1: Boxplot depicting LDL receptor levels after 3 months in patients with low vs. high delta.*

## Figure B2


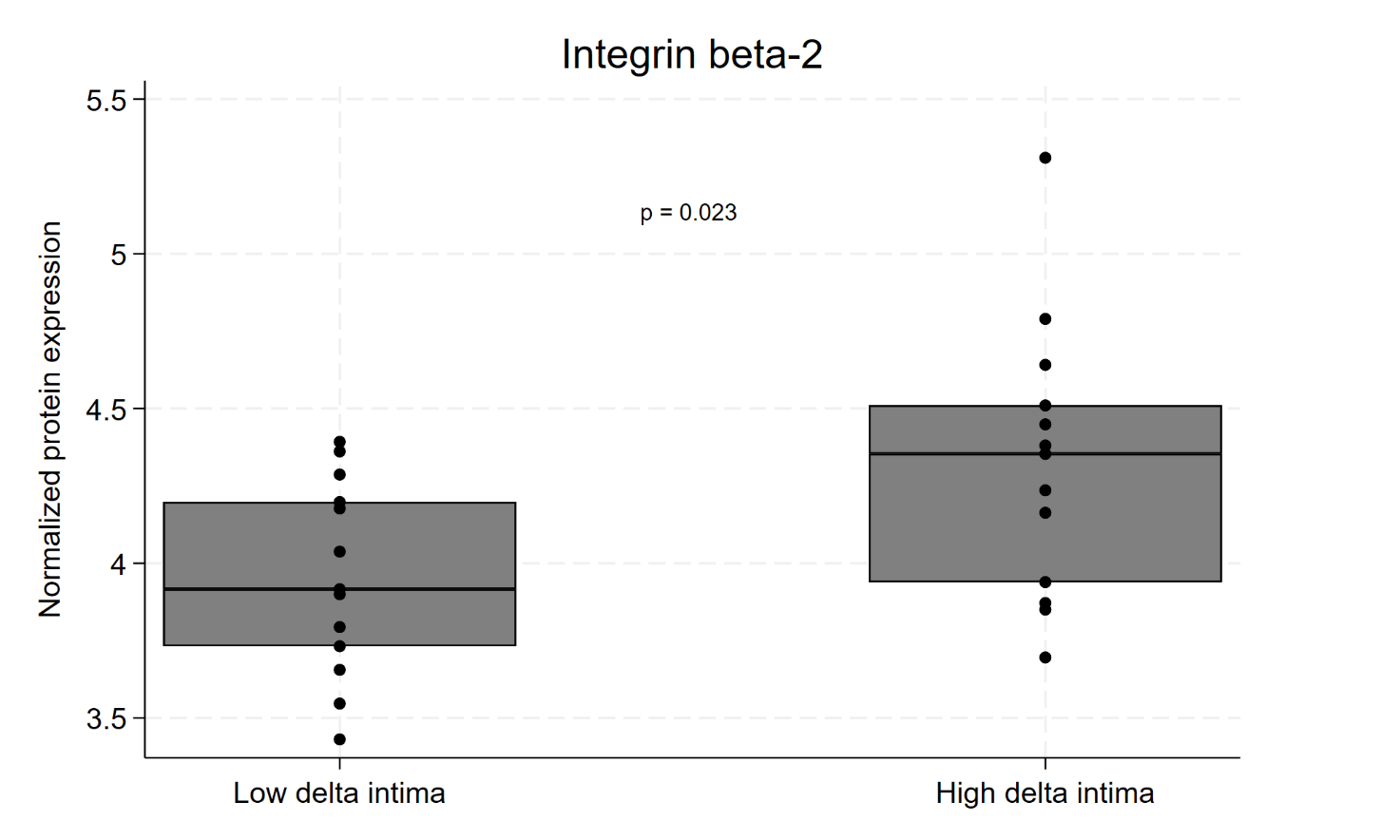


*Figure B2: Boxplot depicting ITGB-2 levels after 3 months in patients with low vs. high delta.*

## Figure B3


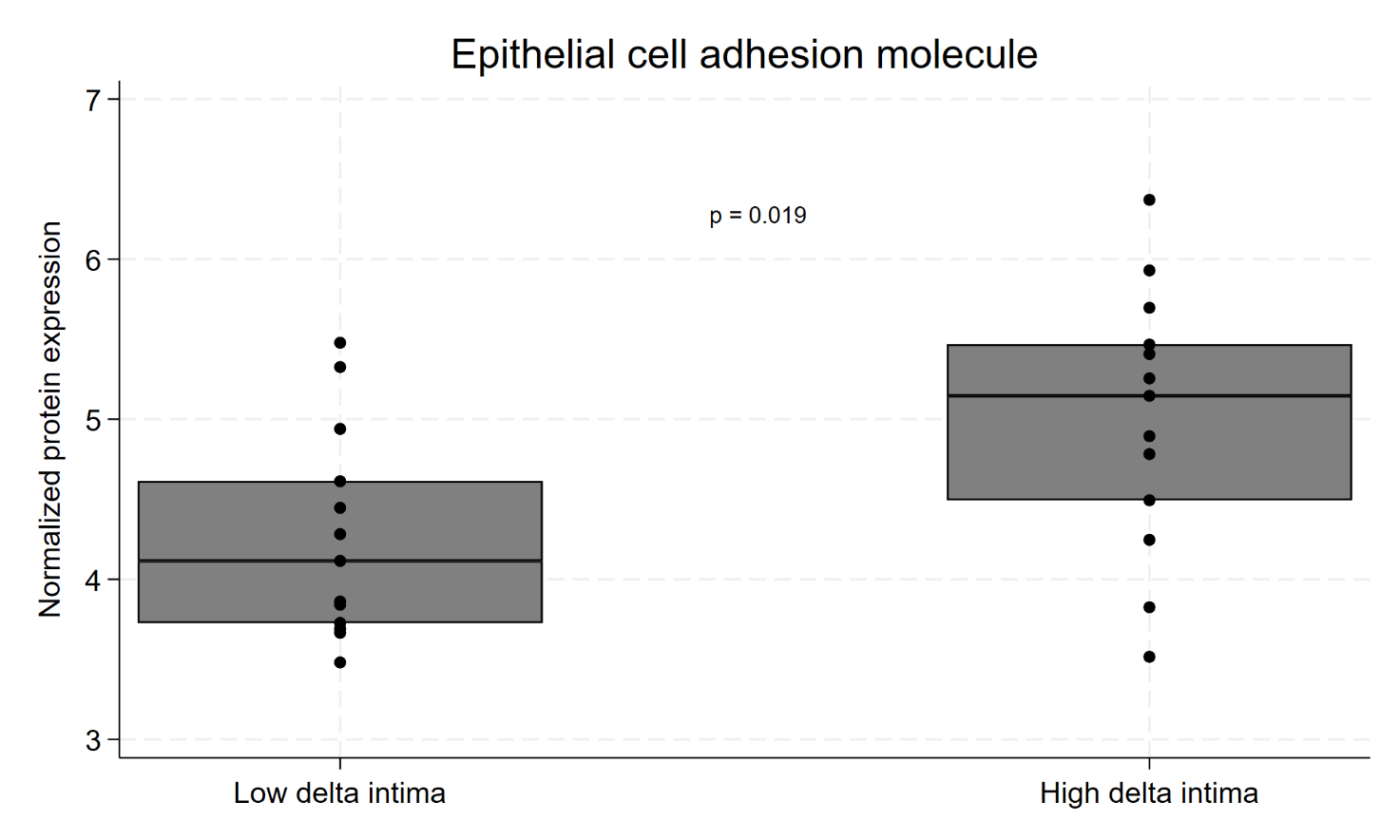


*Figure B3: Boxplot depicting Ep-CAM levels after 3 months in patients with low vs. high delta.*

## Figure B4


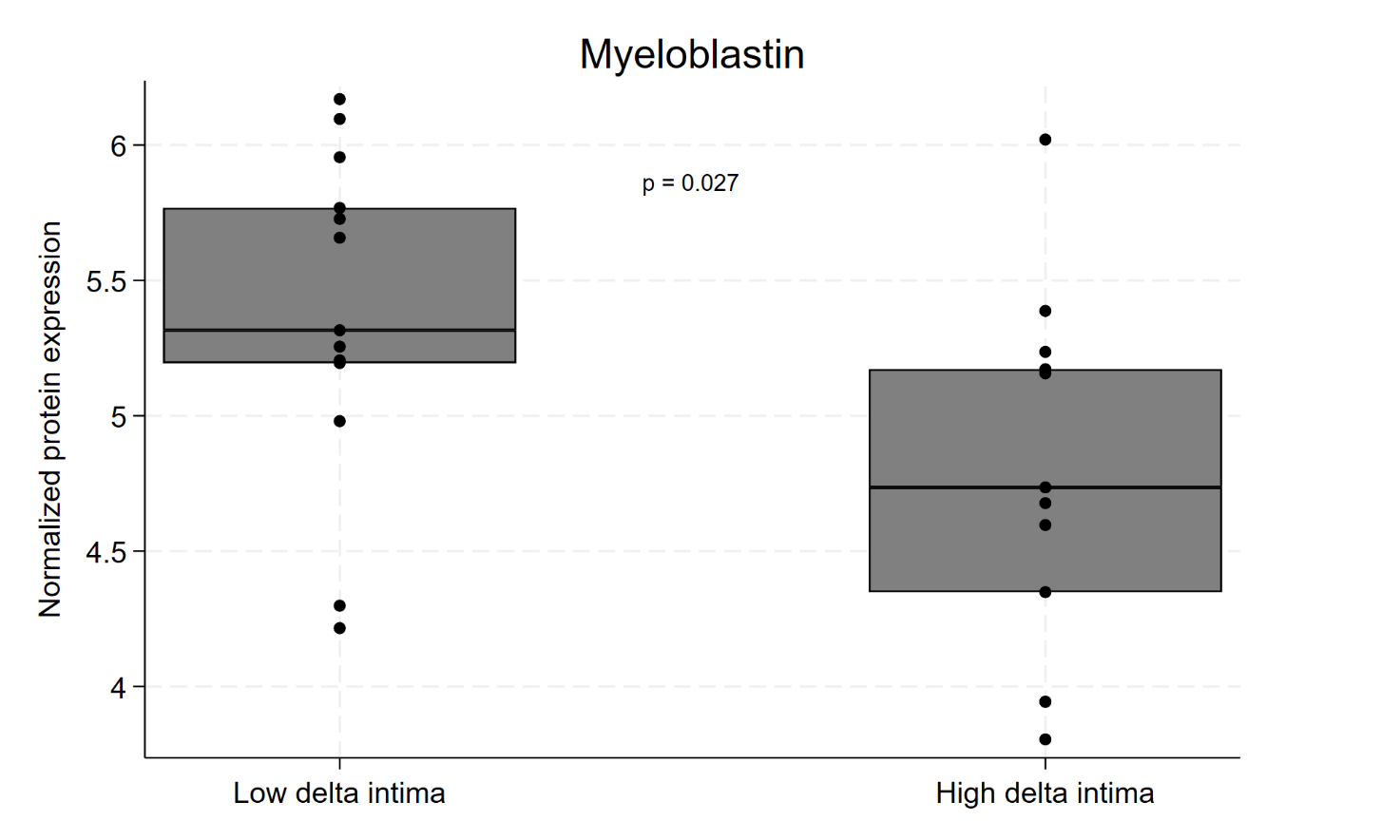


*Figure B4: Boxplot depicting PRTN3 levels after 3 months in patients with low vs. high delta.*

## Figure B5


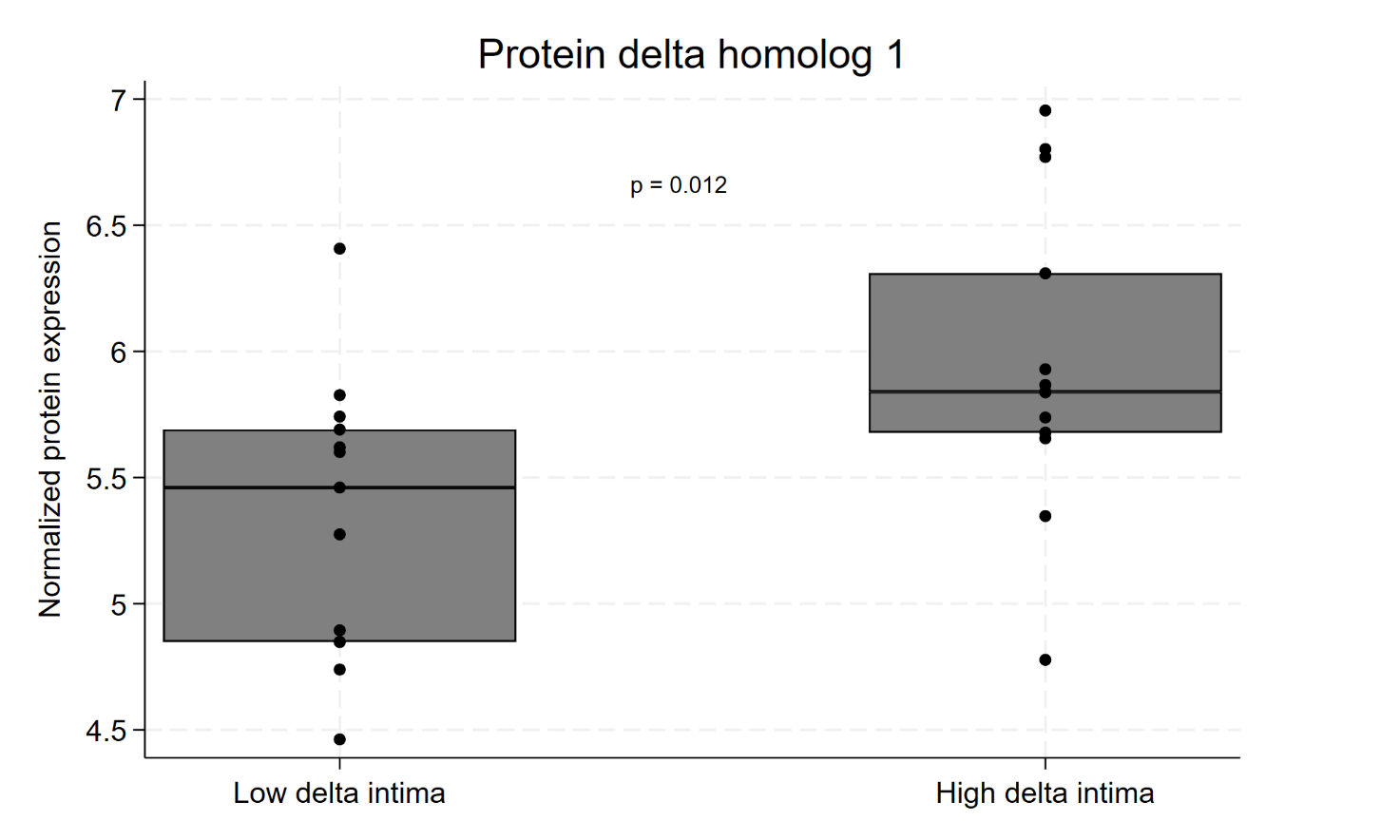


*Figure B5: Boxplot depicting DLK-1 levels after 12 months in patients with low vs. high delta.*

## Figure B6


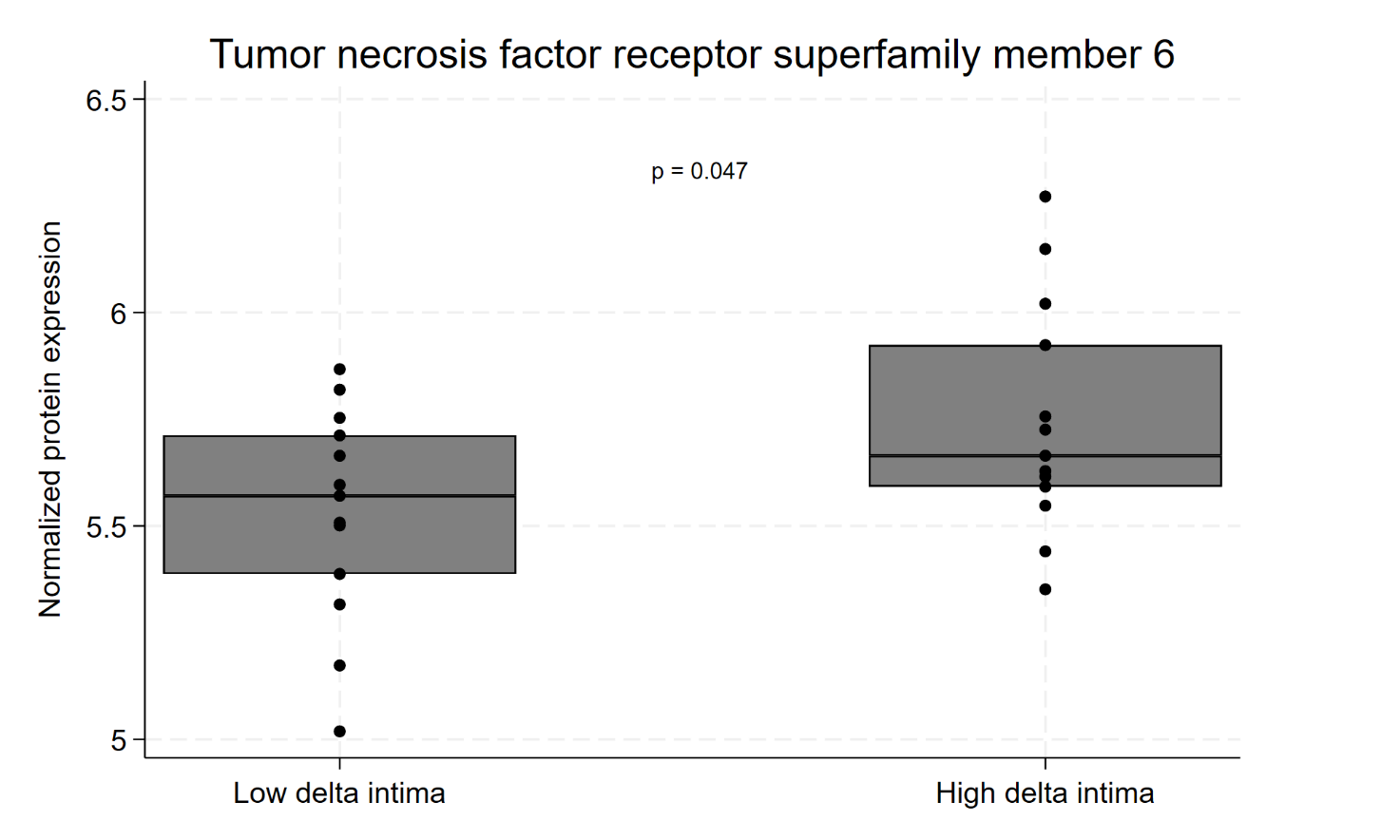


*Figure B6: Boxplot depicting FAS levels after 12 months in patients with low vs. high delta.*

## Figure B7

*
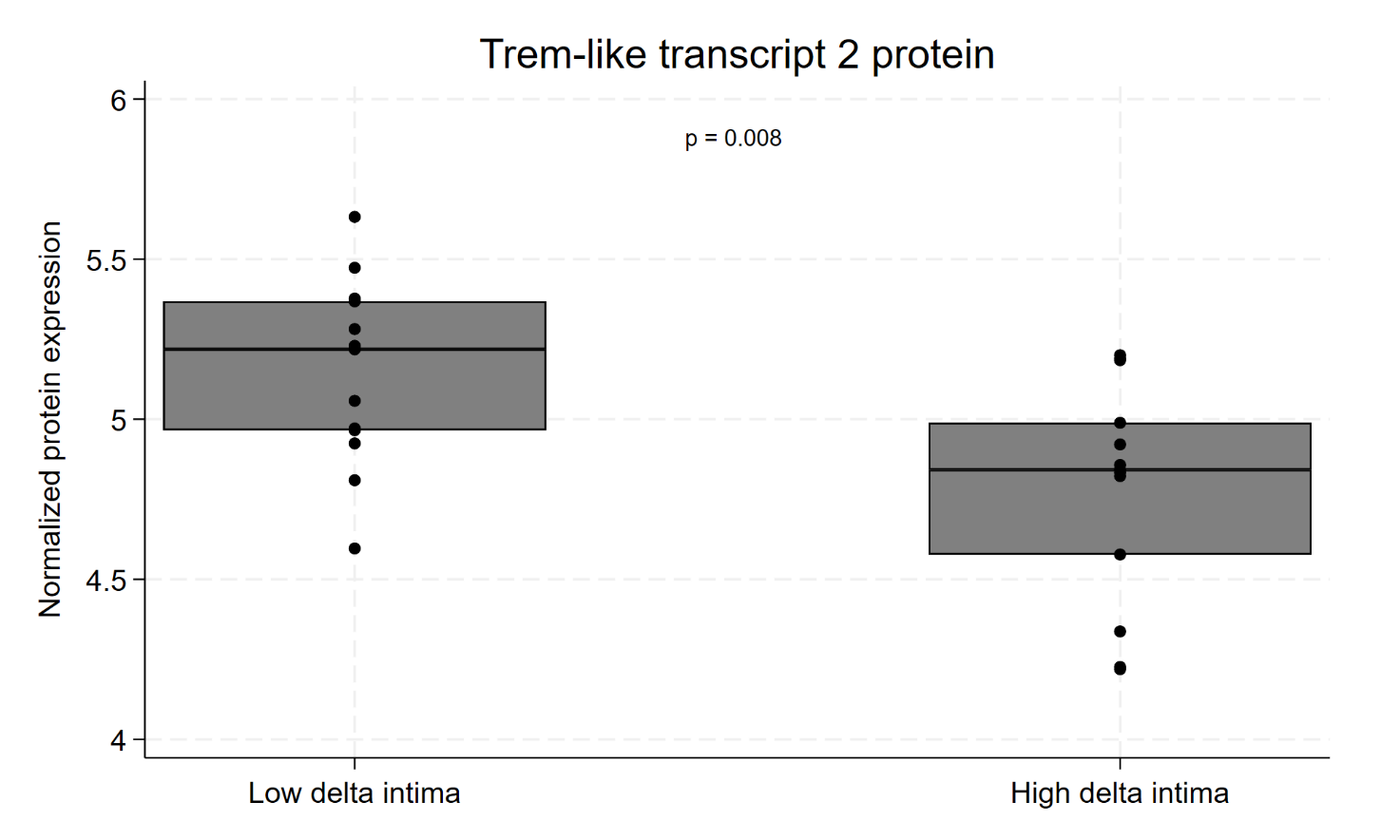
 Figure B7: Boxplot depicting TLT-2 levels after 12 months in patients with low vs. high delta.*

## Figure B8

*
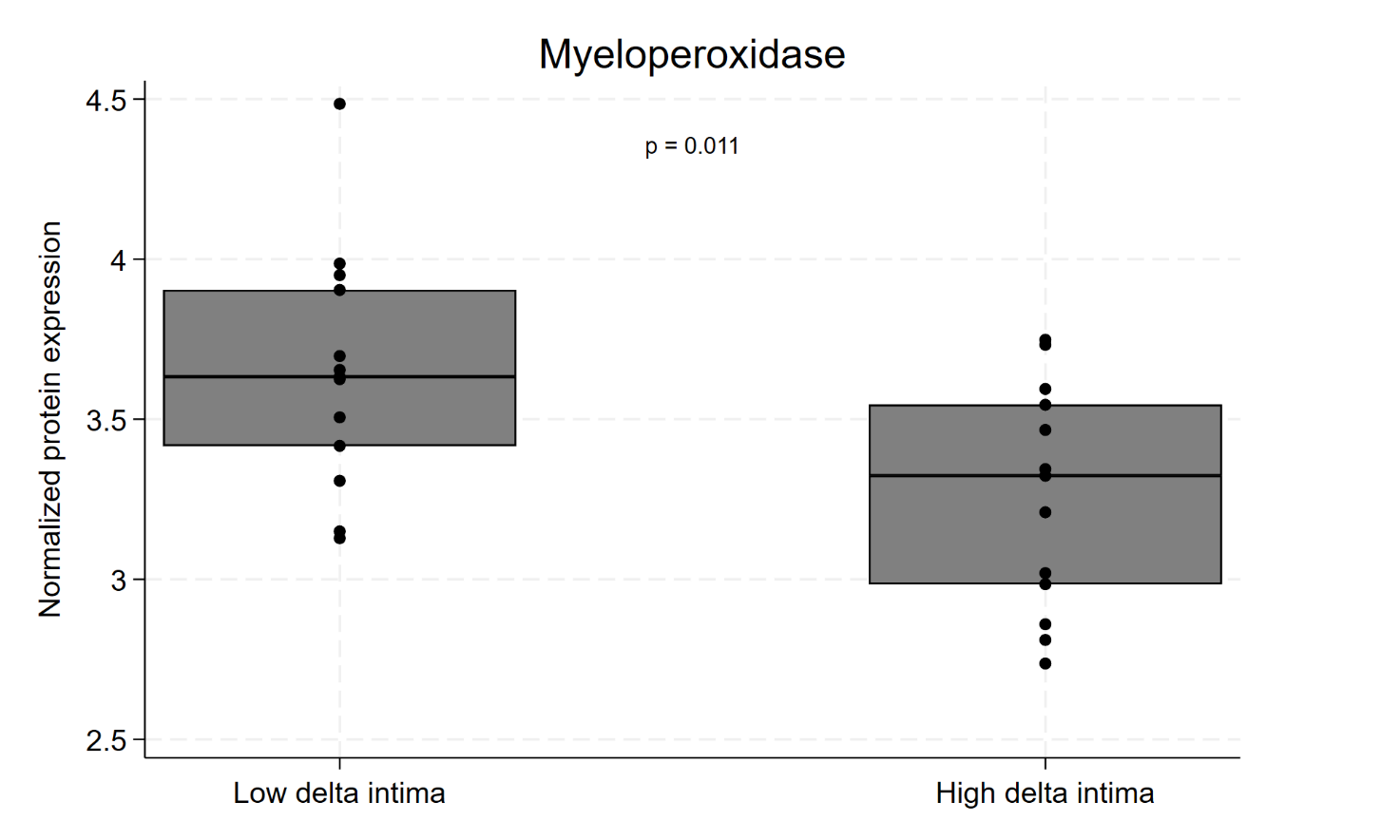
 Figure B8: Boxplot depicting MPO levels after 12 months in patients with low vs. high delta.*

## Figure B9

*
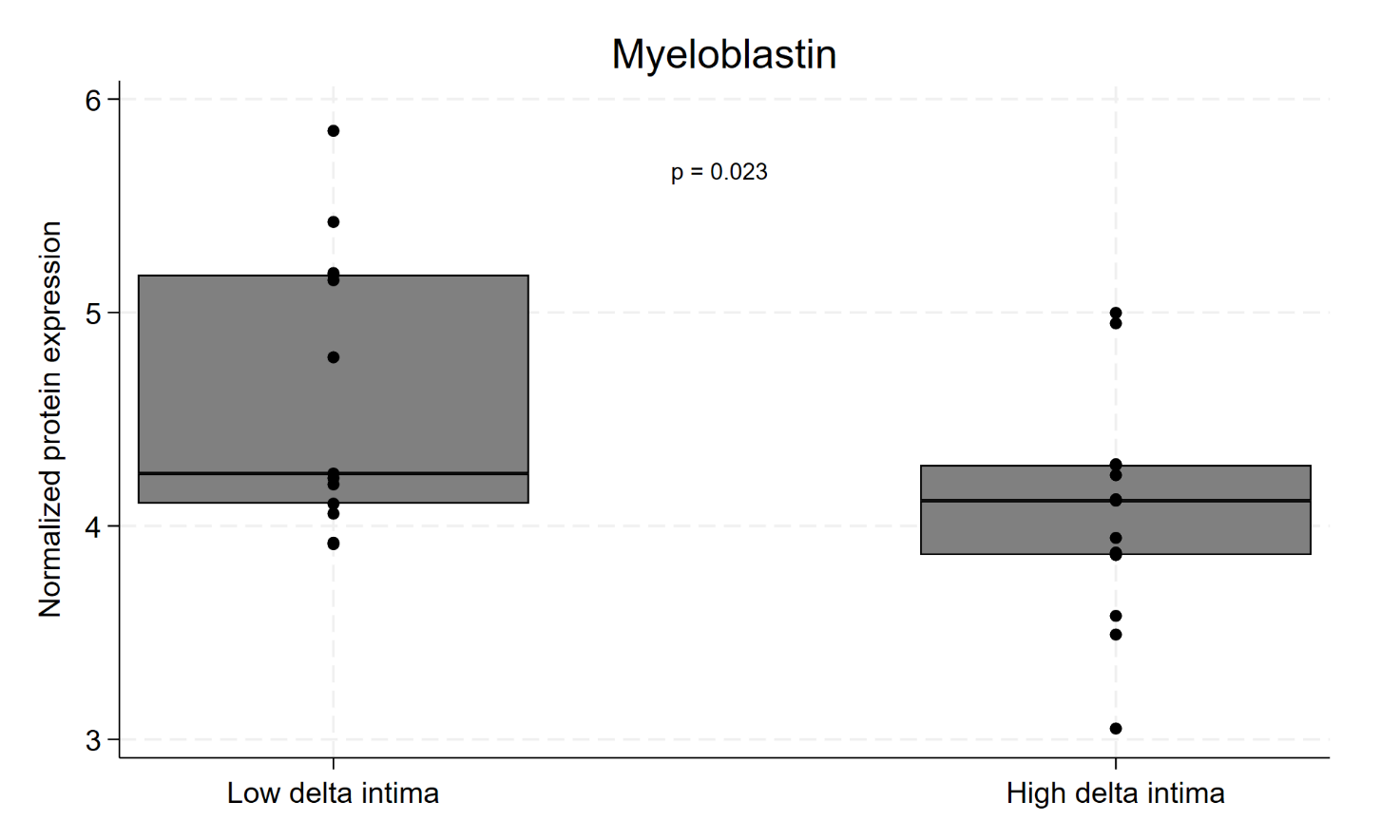
 Figure B9: Boxplot depicting PRTN3 levels after 12 months in patients with low vs. high delta.*

## Figure B10

*
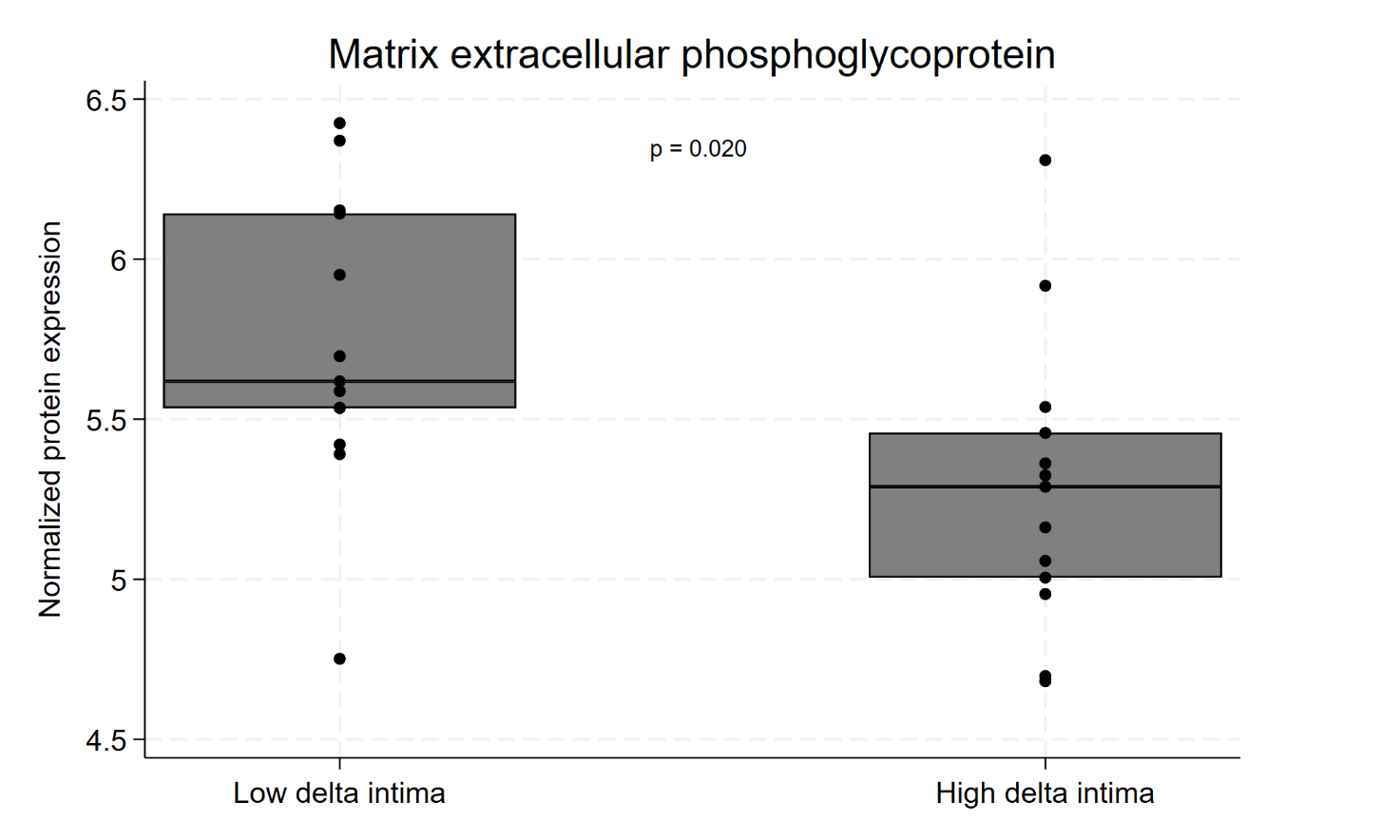
 Figure B10: Boxplot depicting MEPE levels after 12 months in patients with low vs. high delta.*

## Figure B11

*
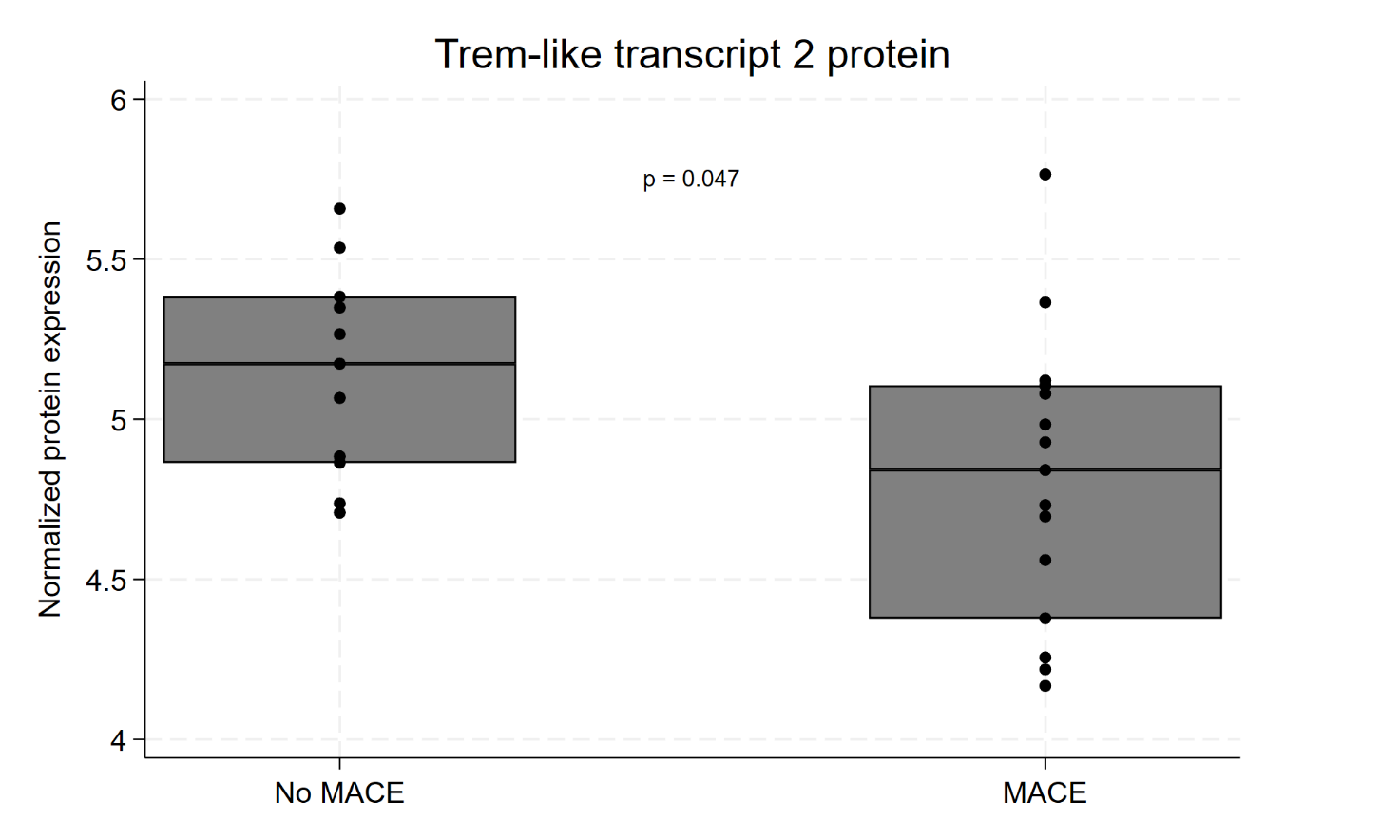
 Figure B11: Boxplot depicting TLT-2 levels after 3 months in patients experiencing MACE vs. no MACE.*

## Figure B12

*
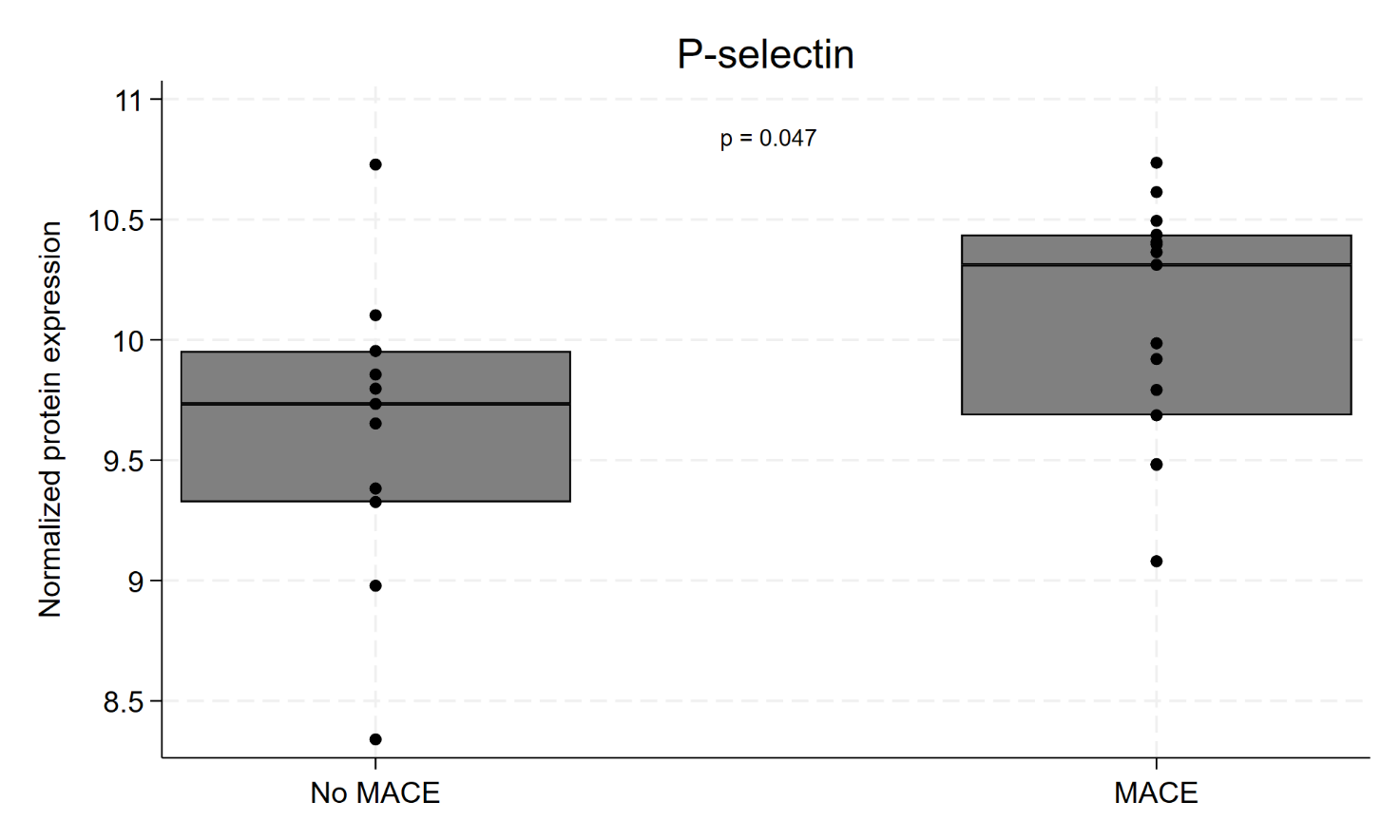
 Figure B12: Boxplot depicting SELP levels after 12 months in patients experiencing MACE vs. no MACE.*

## Figure B13

*
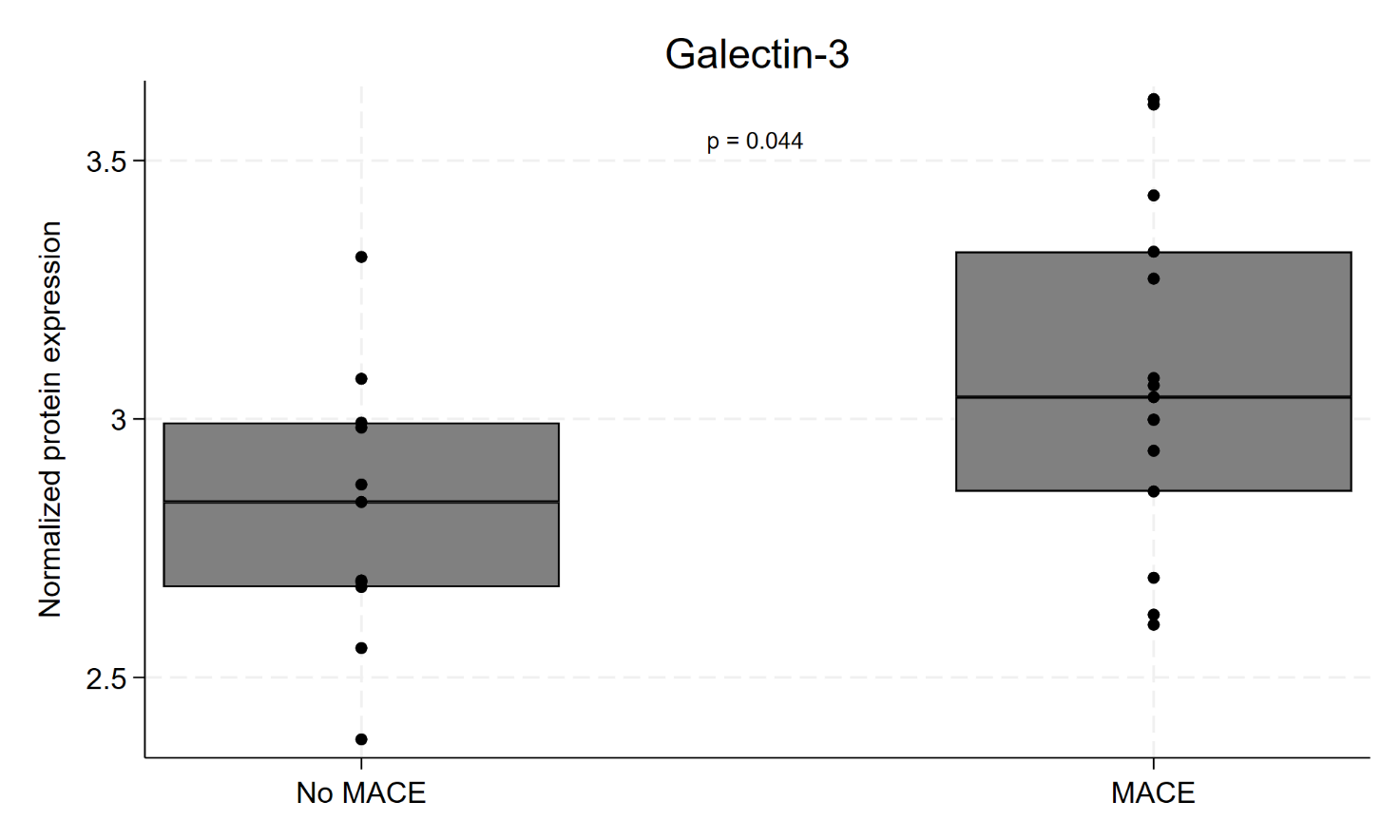
 Figure B13: Boxplot depicting Gal-3 levels after 12 months in patients experiencing MACE vs. no MACE.*

## Figure B14

*
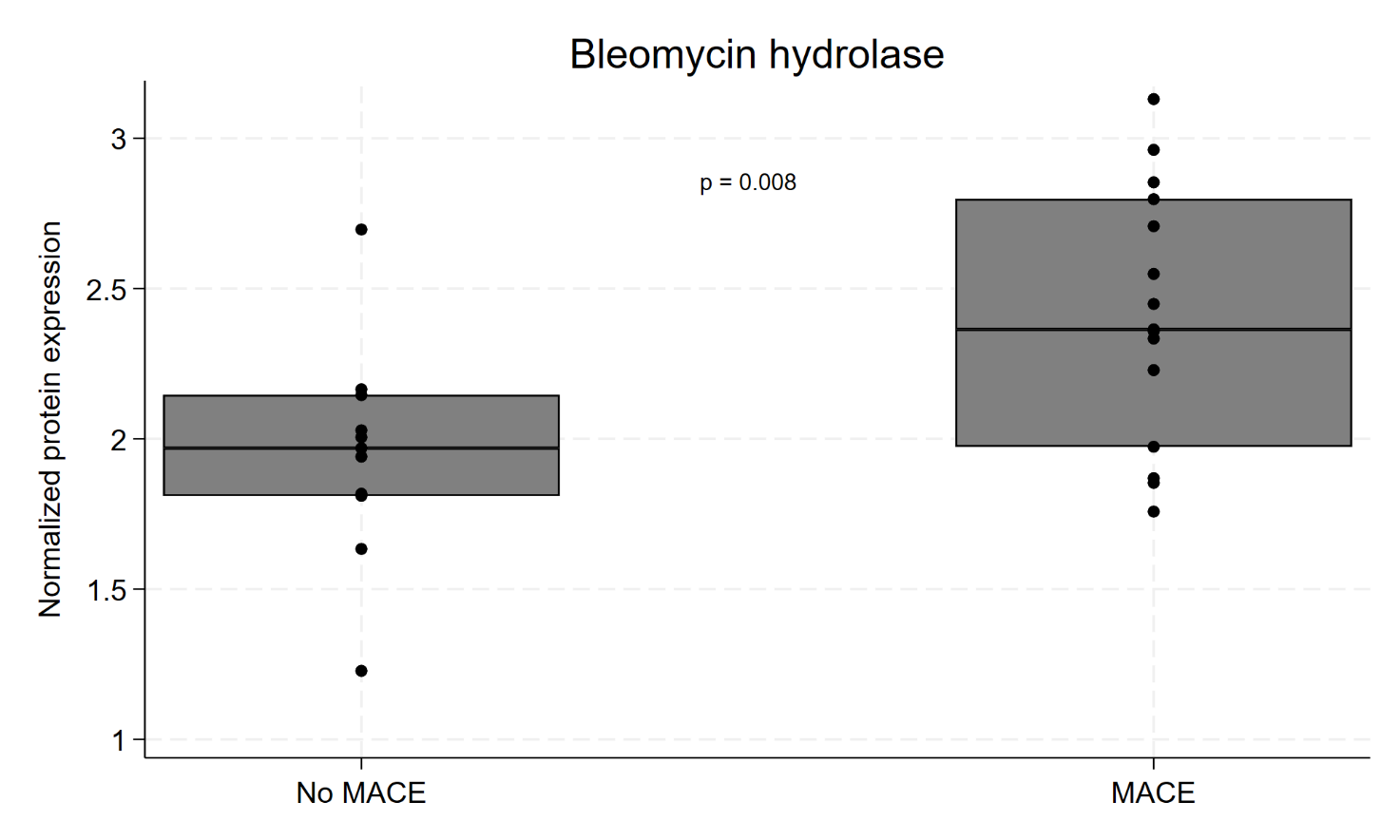
 Figure B14: Boxplot depicting BLM hydrolase levels after 12 months in patients experiencing MACE vs. no MACE.*

## Figure B15

*
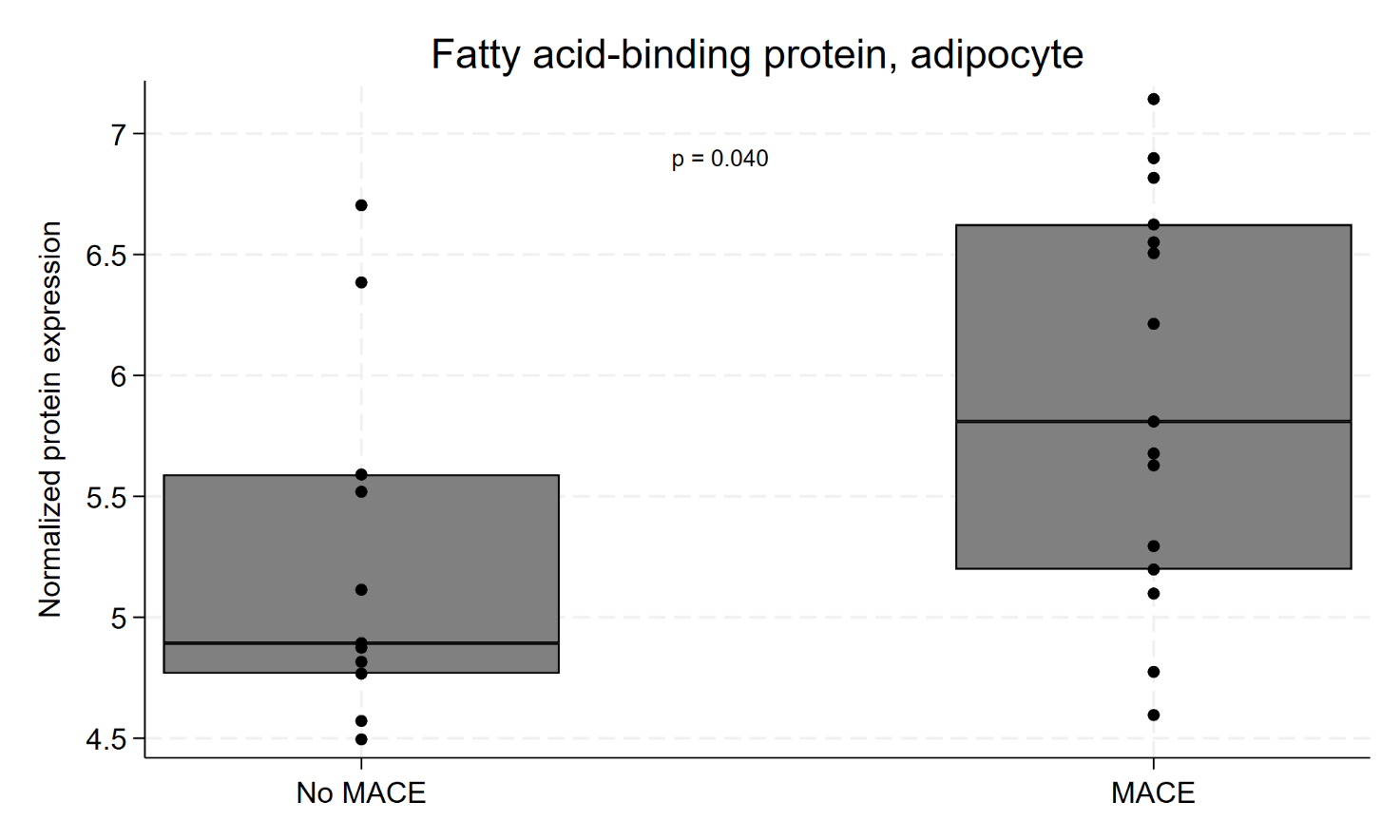
 Figure B15: Boxplot depicting FABP4 levels after 12 months in patients experiencing MACE vs. no MACE.*

## Figure B16

*
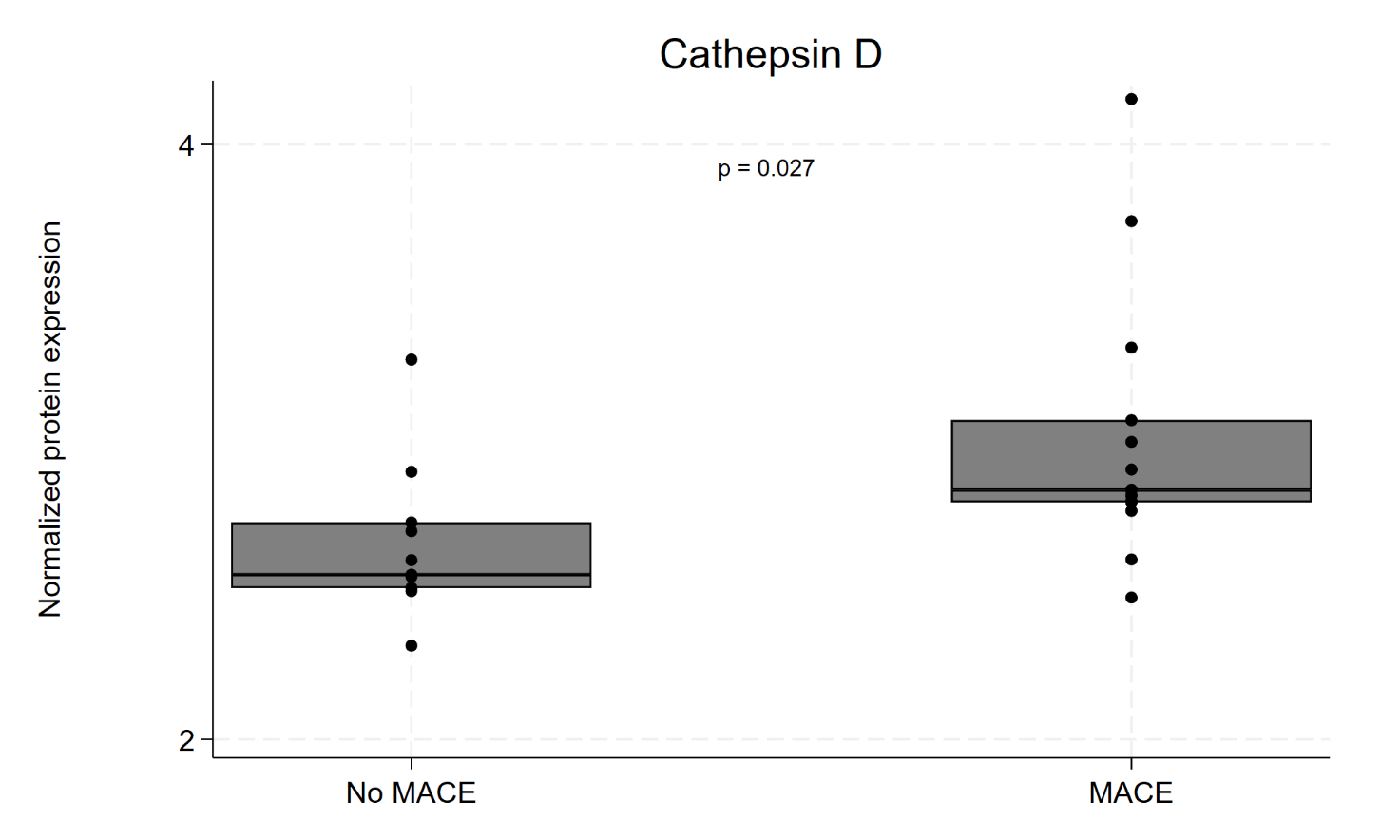
 Figure B16: Boxplot depicting CTSD levels after 12 months in patients experiencing MACE vs. no MACE.*

## Figure B17

*
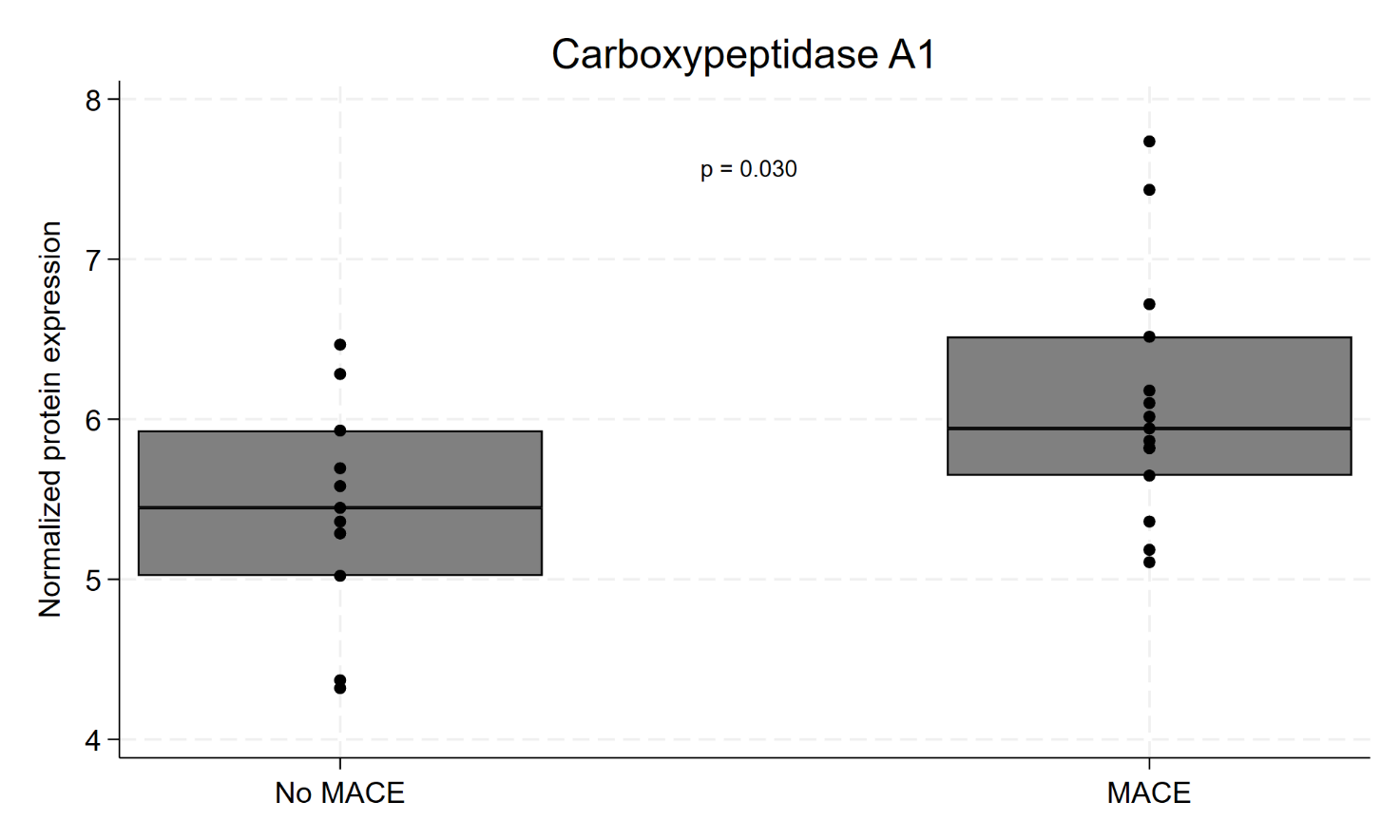
 Figure B17: Boxplot depicting CPA1 levels after 12 months in patients experiencing MACE vs. no MACE.*

## Figure B18

*
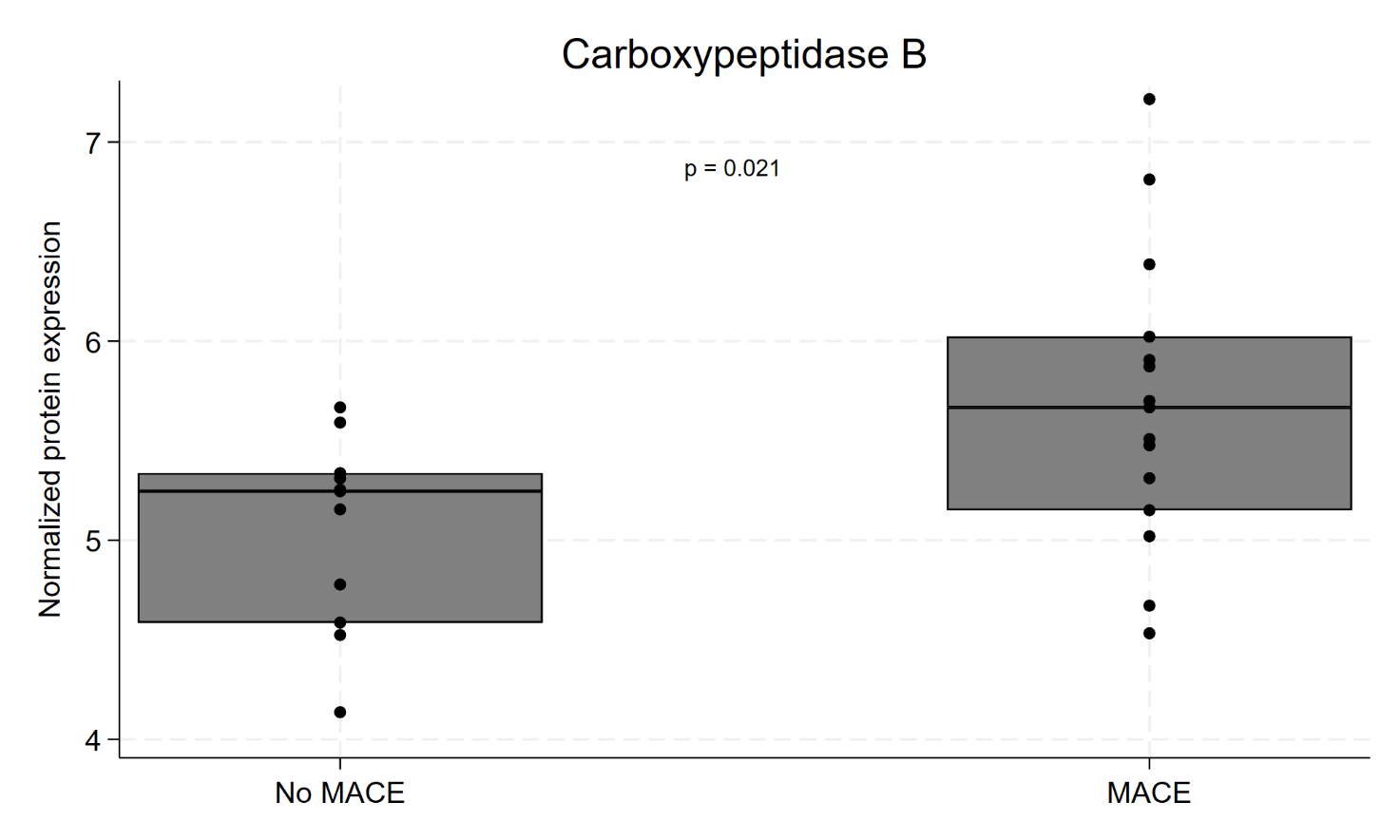
 Figure B18: Boxplot depicting CPB1 levels after 12 months in patients experiencing MACE vs. no MACE.*

## Figure B19

*
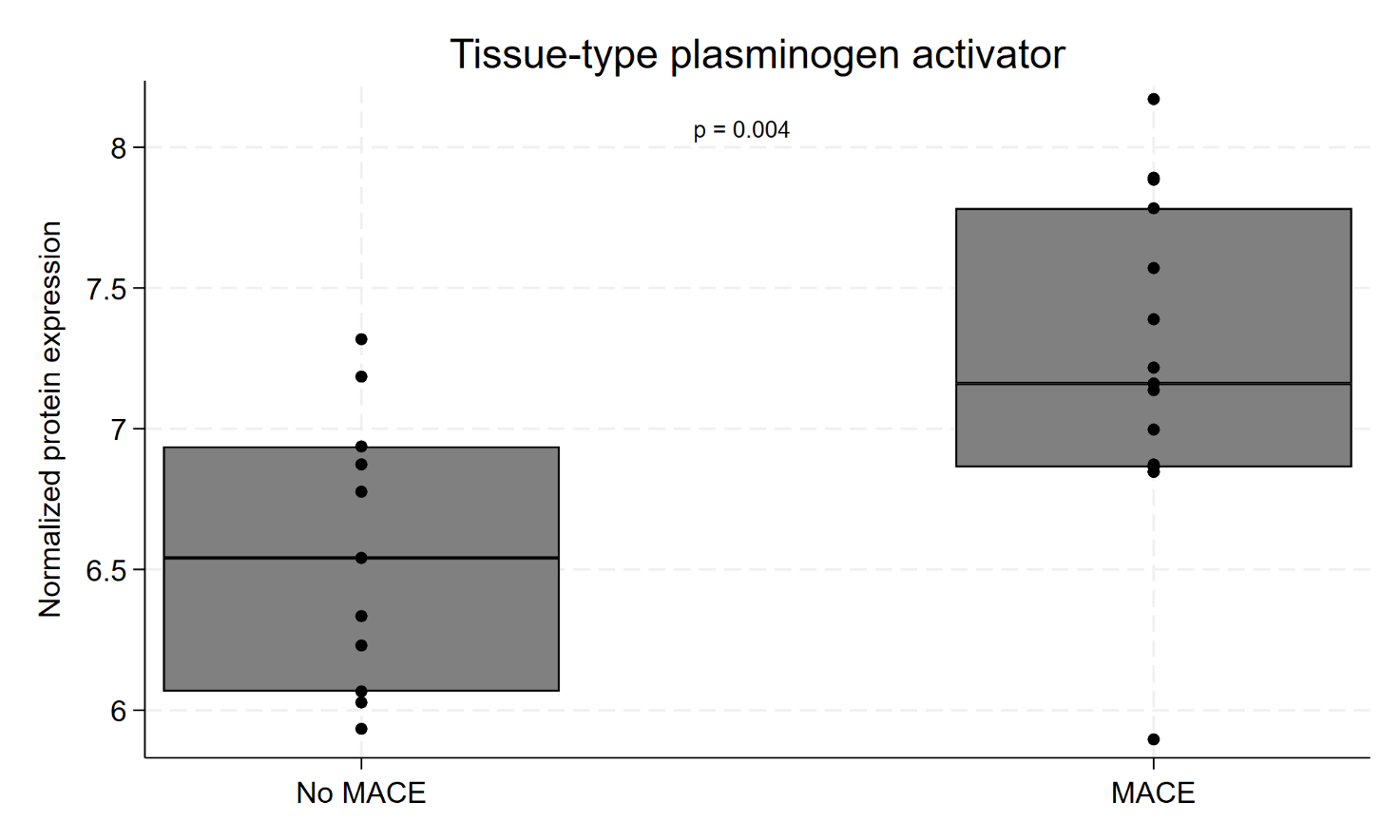
 Figure B19: Boxplot depicting t-PA levels after 12 months in patients experiencing MACE vs. no MACE.*

## Figure B20

*
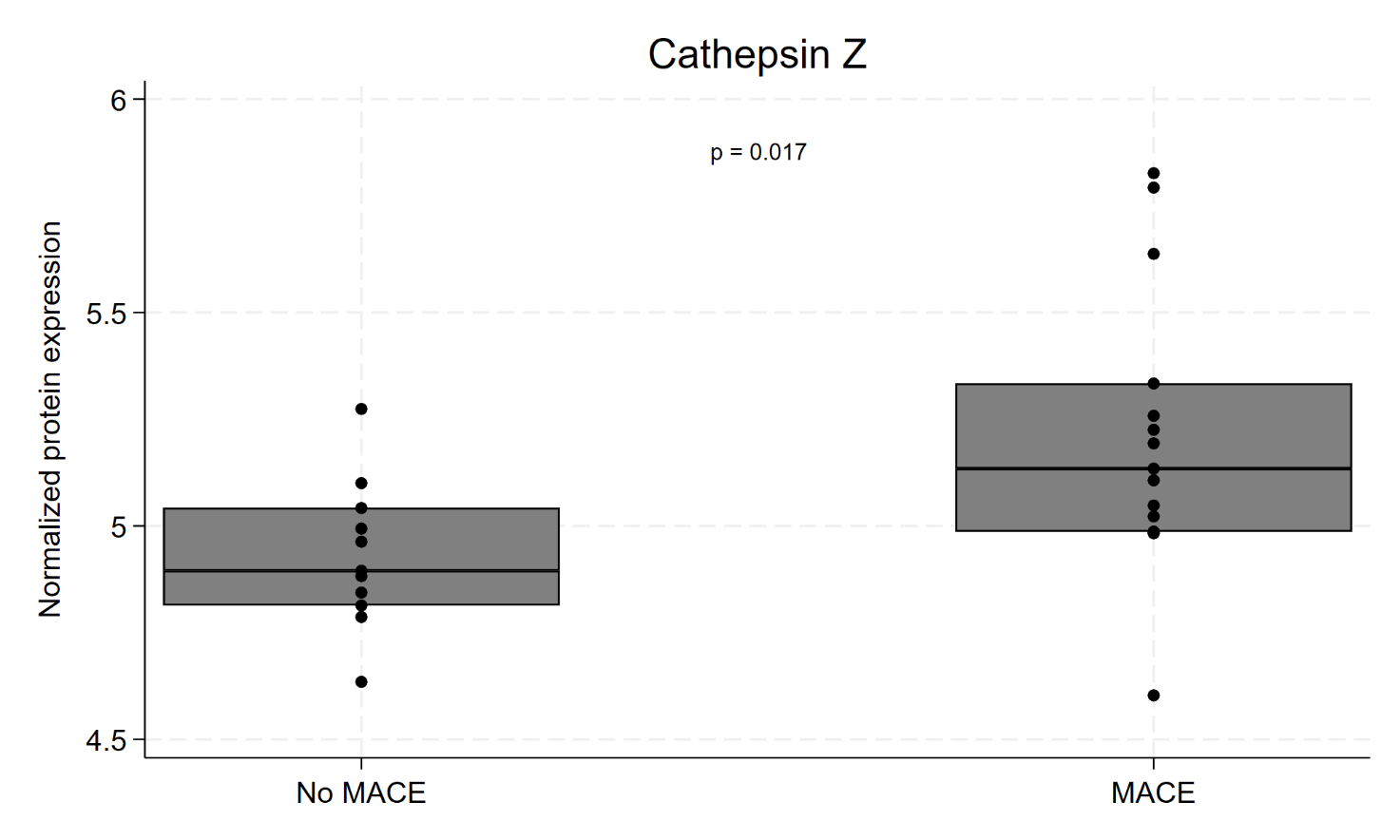
 Figure B20: Boxplot depicting CTSZ levels after 12 months in patients experiencing MACE vs. no MACE.*

## Figure B21

*
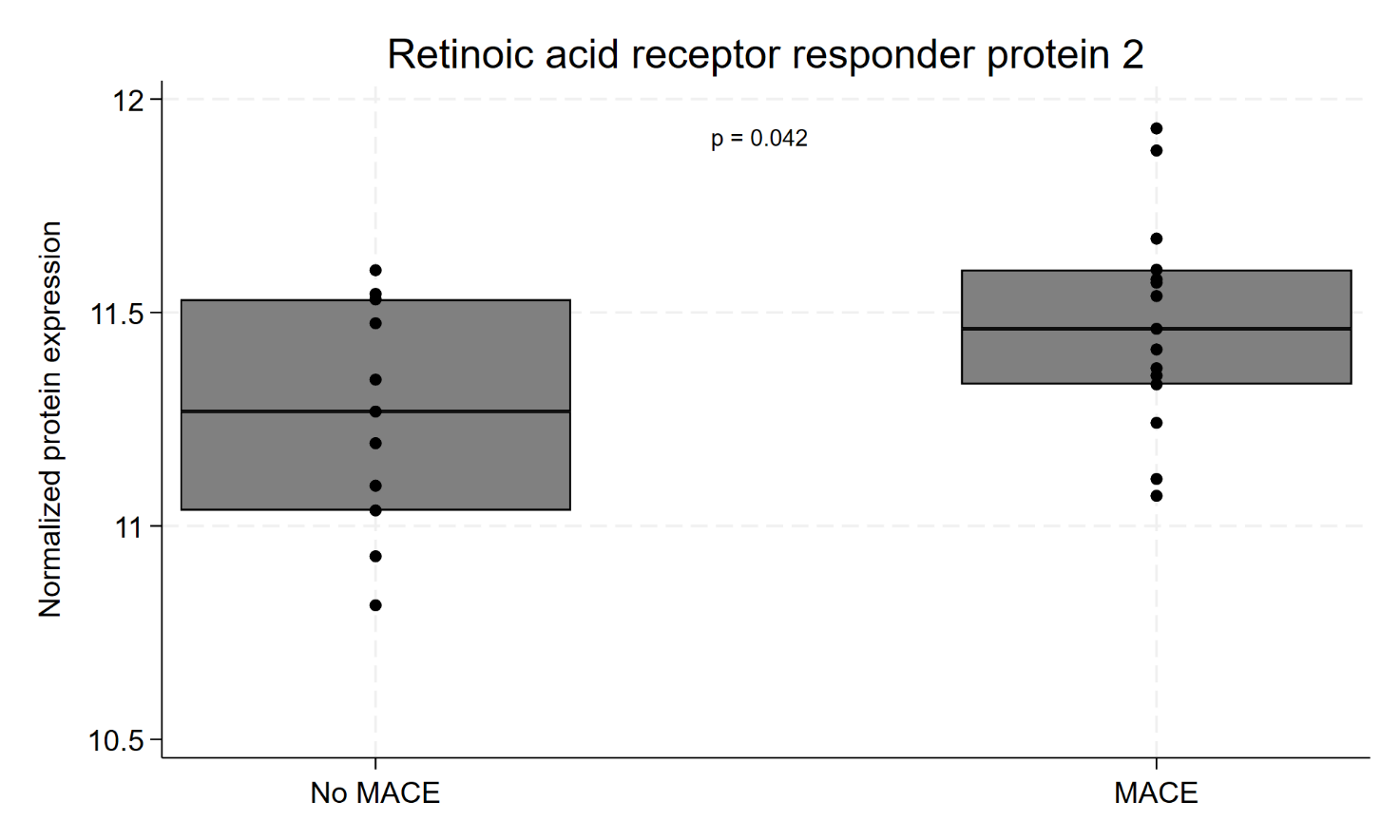
 Figure B21: Boxplot depicting RARRES2 levels after 12 months in patients experiencing MACE vs. no MACE.*
